# XML Data and Knowledge-Encoding Structure for a Web-Based and Mobile Antenatal Clinical Decision Support System: Development Study

**DOI:** 10.2196/17512

**Published:** 2020-10-16

**Authors:** Ever Augusto Torres Silva, Sebastian Uribe, Jack Smith, Ivan Felipe Luna Gomez, Jose Fernando Florez-Arango

**Affiliations:** 1 Bioengineering Research Group Universidad Pontificia Bolivariana Medellin Colombia; 2 Department of Microbial Pathogenesis and Immunology Texas A&M Unversity College Station, TX United States; 3 Department of Humanities in Medicine Texas A&M University Bryan, TX United States

**Keywords:** clinical decision support systems, computer-interpretable guidelines, knowledge representation, state machine, system design, XML

## Abstract

**Background:**

Displeasure with the functionality of clinical decision support systems (CDSSs) is considered the primary challenge in CDSS development. A major difficulty in CDSS design is matching the functionality to the desired and actual clinical workflow. Computer-interpretable guidelines (CIGs) are used to formalize medical knowledge in clinical practice guidelines (CPGs) in a computable language. However, existing CIG frameworks require a specific interpreter for each CIG language, hindering the ease of implementation and interoperability.

**Objective:**

This paper aims to describe a different approach to the representation of clinical knowledge and data. We intended to change the clinician’s perception of a CDSS with sufficient expressivity of the representation while maintaining a small communication and software footprint for both a web application and a mobile app. This approach was originally intended to create a readable and minimal syntax for a web CDSS and future mobile app for antenatal care guidelines with improved human-computer interaction and enhanced usability by aligning the system behavior with clinical workflow.

**Methods:**

We designed and implemented an architecture design for our CDSS, which uses the model-view-controller (MVC) architecture and a knowledge engine in the MVC architecture based on XML. The knowledge engine design also integrated the requirement of matching clinical care workflow that was desired in the CDSS. For this component of the design task, we used a work ontology analysis of the CPGs for antenatal care in our particular target clinical settings.

**Results:**

In comparison to other common CIGs used for CDSSs, our XML approach can be used to take advantage of the flexible format of XML to facilitate the electronic sharing of structured data. More importantly, we can take advantage of its flexibility to standardize CIG structure design in a low-level specification language that is ubiquitous, universal, computationally efficient, integrable with web technologies, and human readable.

**Conclusions:**

Our knowledge representation framework incorporates fundamental elements of other CIGs used in CDSSs in medicine and proved adequate to encode a number of antenatal health care CPGs and their associated clinical workflows. The framework appears general enough to be used with other CPGs in medicine. XML proved to be a language expressive enough to describe planning problems in a computable form and restrictive and expressive enough to implement in a clinical system. It can also be effective for mobile apps, where intermittent communication requires a small footprint and an autonomous app. This approach can be used to incorporate overlapping capabilities of more specialized CIGs in medicine.

## Introduction

### Background and Significance

With the increasing adoption of electronic health records and hospital information systems, the implementation of clinical practice guidelines (CPGs) through integration with these information systems is possible during clinical encounters [1], creating what is known as clinical decision support systems (CDSSs), which assist physicians during health care encounters. CDSSs attempt to mimic the way humans use clinical guidelines with patient information and make decisions based on existing clinical knowledge and knowledge specific to a patient [2]. These systems are built using representations of knowledge and information about different diseases, treatment protocols, findings, and interpretations [3].

A CDSS produces patient-specific output based on patient data combined with these representations of medical knowledge [4]. Traditionally, three functions are supported by a CDSS: the provision of automated clinical information management such as data entry and retrieval, the attention-focusing functions such as medical alerts and reminders, and the provision of patient-specific recommendations or advice based on individual patient data [5]. All these functions can be useful in providing health care services, but the execution and effectiveness of these functions are determined by the effectiveness of the utilization of medical knowledge in the associated clinical workflow [3].

There have been widespread reports of dissatisfaction and user complaints with existing CDSS functionality, and satisfaction is considered one of the primary challenges for improving CDSS development and acceptance [6]. A special challenge in this regard is implementing a CDSS system with interactions that match actual and desired clinical workflow as closely as possible. This requires that the CDSS interactions with the user be context sensitive and accessible at the point of care [7]. More importantly, studies have shown that a CDSS should also integrate into the overall organizational workflow in order to make its use easy and efficient [4].

In a CDSS, computer-interpretable guidelines (CIGs) are used to formalize medical knowledge contained in CPGs into a computable form. Additionally, we can create a set of software functions for the CDSS user interactions that also match the clinical workflow [6]. However, existing CIG frameworks require a specific interpreter or compiler for each computable representation language, hindering their widespread implementation and interoperability [7]. This has led us to propose a different approach to CIGs and workflow representation that addresses how both clinical knowledge and data are represented while retaining the ability to capture workflow constraints in order to create a more positive user perception of the CDSS. We also desired to maintain a small software and communications footprint for web applications and mobile apps. Our approach was originally intended to create a readable and minimal syntax for a web and mobile application CDSS for antenatal care guidelines that would improve human-computer interaction and enhance usability by aligning the system behavior with clinical workflow. In the antenatal system design, a requirement was to implement a CDSS that was well integrated with existing medical workflow, first as a web application and then as a mobile app. A number of such systems have been developed and shown to improve clinical outcomes [8-10].

### Alternative Solutions

We considered several different representation languages for our CDSS system. One approach we considered was to use an existing CIG syntax, such as Arden syntax or Guideline Interchange Format, to represent the medical knowledge found in the CPG. However, we decided to develop our own syntax to handle clinical data and knowledge because we are aiming to implement our CDSS with a model-view-controller (MVC) architecture appropriate for both web applications and mobile apps. We intended to repurpose the CIG knowledge engine core and the represented medical content for mobile app development. We had the additional goal of ensuring that the executable representation be human readable and have sufficient but minimal syntax and process elements for our particular CPGs. Importantly, we took a minimal design approach, in that we wanted the footprint of the software to have as small a computational and data footprint as possible, since we intended to use the resulting tools both in a web application and mobile app. In our environment, the mobile app may not have continuous connectivity to the networked computing resources, requiring it to execute autonomously if necessary.

With these requirements in mind, we chose XML as a better language to represent CPG knowledge. For web applications and mobile apps, this is an ideal choice as a core component of the World Wide Web. XML is key to formatting content into HTML pages and is an industry standard for data communication among different computer systems [11]. We took advantage of the XML schema over other formats like JavaScript Object Notation to validate the computable representation of CPG documents and verify each piece of item content in a document [12].

There is increasing interest in software frameworks and the feasibility of representing knowledge such as CPGs in semantic web technologies [13-18]. One such advanced system combines a formalization of CPGs using fuzzy cognitive maps (FCMs) implemented in semantic web technology [10]. The CPGs are represented in the form of if-then fuzzy rules. The representation of FCMs uses Notation 3 [11], which is a shorthand non-XML serialization of resource description framework models. The Euler sharp reasoning engine [12] is used to implement inferencing for this FCM implementation. This approach, as well as similar ones that use a sematic web technology layer, has great expressive power but is in conflict with our minimalist requirements for mobile environments, in which connectivity is intermittent and thus requires that CDSS run with minimal computing resources without connectivity to the internet.

There are many other challenges to CDSSs in the environments in which we are implementing our system. There are issues of scale in the management of guidelines in CIG forms, history tracking, CIG version control, and automatic aggregation of CIGs. In this paper, we focus on a narrow subset of such desirable requirements. We are only concerned with having sufficient expressivity of the representation while maintaining a small communication and software footprint for mobile apps that can also be repurposed without modification for incorporation into web applications and other mobile apps. To our knowledge, none of the approaches taken above would be satisfactory for those requirements. In the future, as our approach develops, we will also address these other important issues and be in a better place to compare our approach on those dimensions. In this paper, we focus on the adequacy of satisfying the requirements for expressivity and minimal communication and computing resources.

**Figure 1:**
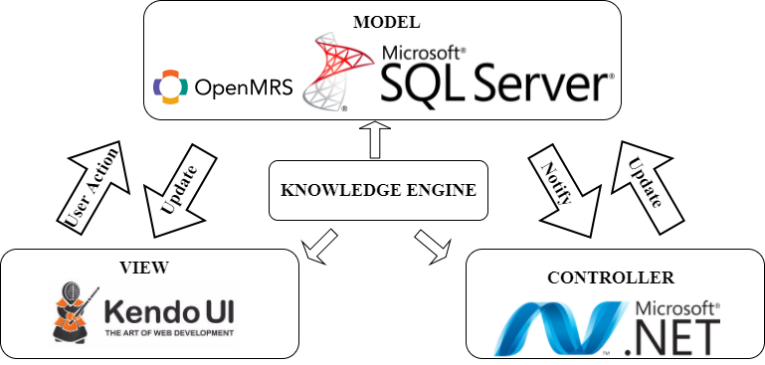
Architecture design used in the web-based clinical decision support system.

In Figure 1, we illustrate the architecture design of our CDSS, which uses the MVC architecture and a knowledge engine based on XML. Specifically, we have (1) a model, (2) a view, (3) a controller, and (4) a knowledge engine.

First, the model consists mainly of a database system used to store the patient's phenotypic and laboratory data so the database can be queried when required [19]. In our CDSS web application, we use Microsoft SQL Server (Microsoft Corp) as the relational database management system with a data model based on the OpenMRS (OpenMRS Inc) medical record system.

Second, the view refers to the system components the user interacts with that display in the user interface.

Third, the controller refers to the system components responsible for processing queries and the user’s interactions. The controllers pass patient input data to the computable guideline model, which is used to query the database, and then select a view to display for the user interface [19].

Fourth, the knowledge engine is the CIG where the decision-making rules capturing the CPGs are stored and where the knowledge for integrating the clinical health care workflow and CPGs are stored and interpreted.

In this paper, we exclusively present the design of the knowledge engine, a collection of XML documents and an associated interpreter to handle clinical data and encoded knowledge. The relation between the functionality of the CIG subsystem and the MVC architecture involves the knowledge engine driving the controller’s functionality, alongside a graphic representation of the views. We analyzed some CIG syntaxes in order to find common and minimal features for knowledge representation to incorporate in our XML. We particularly wanted to have design control of features directing user attention to system outputs and improve system usability by being able to match the standard workflow of the clinical situation.

## Methods

### Knowledge Engine Background

For the CDSS knowledge engine, we adopted state machines, artificial intelligence (AI) planning techniques and methods, and minimal CIG functionality. We now briefly describe our decision-making process for each of these.

### State Machine Modeling of Behavior for Health Care

There are many ways to computationally model knowledge and behavior in AI systems, and the use of state machines is one of the oldest and best known [14]. State machines model a system’s states, or features of a system, at a particular point in time and characterize its future behavior based on these states [15]. In our approach, we use state machine models as an intermediate level of design and implementation before representing the state machines in executable XML code.

### Planning

In AI, a plan is generally defined as a sequence of actions that will achieve a specific state. A state refers to the multiple logical conditions that are true in a certain situation, or what could be also known as the “state of the world” [16]. Planning can be used to take advantage of the knowledge of the world. Knowledge in AI refers to information and conditions about the world and how actions change and affect the world [17]. AI planning approaches can be used to represent what is known about the current state of the world and the available actions. In terms of our CDSS, the world would be the patient’s situation, and the actions would be the testing, observations, diagnoses, and treatments that change the state of the patient. An AI planning conceptualization of CIG allows for a framework that incorporates the various approaches to CIG taken in Table 1 and our approach. Adopting this approach allows us to envision using AI planning techniques and methods for this part of the CDSS.

**Table 1:** Computer-interpretable guideline formalisms comparison.

Detail	Arden syntax	GLIF^a^	PROforma	Asbru	Eon
Model	Medical logic module	Object-oriented flowchart structured in steps	PROforma task ontology	Plan	Dharma guideline model
Model elements	Maintenance, knowledge, library, resources	Branch, decision, action, patient state steps	Plans, decisions, actions, inquiries	Preferences, intentions, conditions, effects	Scenarios, decisions, actions, activities
Language	- MLMs^b^ are text based (each MLM is encoded as an ASCII^c^ file)	- UML^d^ class diagrams in GLIF3 XML-based syntax - RDF^e^ language	- Guidelines are translated into language called LR2L - Contains a formal expression language	- DTD^f^ in Backus-Naur form - Control-flow language are defined by means of XML	RDF

^a^GLIF: Guideline Interchange Format.

^b^MLM: medical logic module.

^c^ASCII: American Standard Code for Information Interchange.

^d^UML: Unified Modeling Language.

^e^RDF: Resource Description Framework.

^f^DTD: document type definition.

### Minimal CIG Functionality Objective

In designing a minimal-specification XML file to manage clinical data and knowledge for a web-based and mobile CDSS for antenatal care, we compared and analyzed some CIG approaches found in OpenClinical [20] (see “CIG Comparisons”). We will not attempt to explain each of the methods here, as those details can be found in the original papers [21-25]. We incorporated into our representation the elemental model structures and language of each CIG.

The knowledge engine design also integrated the requirement of matching care workflow that was desired in the CDSS. Here we used a work ontology analysis [26] of the CPG, as it is applied to antenatal care in our particular target clinical settings. The ontology analysis was used to reach a common understanding of the structure of information [27] (see “Knowledge Extraction”) in the care process, since the format of CPGs is not standardized and shows variations according to the organization producing the guidelines and the clinical area [28]. This knowledge extraction and ontology analysis were performed for antenatal health care from published clinical practice guidelines related to pregnancy and childbirth in various sources [1,29-32]. The knowledge and ontology analysis found in the CPGs were then aligned with AI planning theory conceptualizations of the clinical workflow in antenatal care in order to establish the knowledge representations in the knowledge engine.

We then used Microsoft Visual Studio (Microsoft Corp) to develop an XML Schema Definition (XSD) to ease the knowledge-encoding process into rule statements. Lastly, we compared our XML document file with an Arden syntax file that we coded doing a similar task in the interest of comparing our representations with one of the CDSS standards often used to manage and apply clinical knowledge in health care settings.

## Results

### CIGs Comparison

Table 1 displays the key elements of some guideline formalism models. All the approaches support a basic AI planning structure to handle decisions and actions based on medical criteria. At a conceptual level, they are very similar, but the formalisms share similarities and differences in form and terminology. They all abstractly incorporate the AI conceptualization of planning as being capturable by a hierarchical task structure, that is, steps in a plan that can be represented by representations of their preconditions, actions, and goals. The goal itself refers to the values of states of the world or knowledge states that must be attained to satisfy a medical decision criterion that would lead to a certain action. The preconditions specify the context in which an encapsulated collection of represented knowledge (a knowledge module) should be executed, and the action to be taken is typically an action to assist the user (physician or health care worker).

### Knowledge Extraction

As discussed in de Clercq et al [22], a balance must be maintained between the aspects of abstractness, expressiveness, formalization, acquisition, and execution of the knowledge in order to create a successful CIG. Real effectiveness is also dependent on the guideline development and knowledge extraction processes from original sources. We implemented the framework presented in Boxwala et al [33] to extract the knowledge found in the guidelines. We separated the main health care actions into the main actions that health care professionals could perform. Table 2 illustrates some of the results of our ontology and knowledge analysis of the antenatal CPG knowledge. This table is structured into the divisions of knowledge represented in a basic AI planning system of the applicable medical procedures, treatments, diseases, and relevant information in our CPGs. It is important to note that our knowledge representation framework, techniques, and methods were designed in a way that can be a used for other medical system design purposes. The current implementation is a proof of concept that the general framework can be successfully specialized for a specific set of medical problems to be represented in a CDSS.

**Table 2:** Ontology knowledge structure extraction for antenatal clinical practice guidelines.

Main actions	Health care provider action	Goal	Precondition	Action
Observation	Examination findings, family history, lifestyle factors, health summary, pregnancy summary, nutrition summary, physical activity summary, social summary, laboratory test, reason for encounter	Record observation and information about the patient	Information is missing or incomplete	Request information
Evaluation	Absence of information, clinical synopsis, adverse reaction risk, health risk assessment, problem/diagnosis	Analyze information and absences of information	Information is in database	Compare information, risk assessment
Action	Care plan, health education, medication order, laboratory test request, procedure request, notification	Suggest a proper care plan and notify health care provider of an event	Evaluation is done	Suggest medication order, laboratory test, or procedure; generate a notification, alert, or message

### XSD Design

At the next level of system design, we used XML-encoded rules to represent the state transitions of a state machine. This state machine protocol file is the core component for future proposed implementations of CDSS content captured in a state machine and interpreted as state machine behavior.

The CDSS uses XML logical methods to interpret data entries through conditional rules to achieve representations of goals and actions. The XML interpreter will usually execute the logic through a match-and-resolve process. First, all rules with conditions corresponding with the input data are evaluated, and if the conditions are satisfied, the rules will execute.

**Figure 2:**
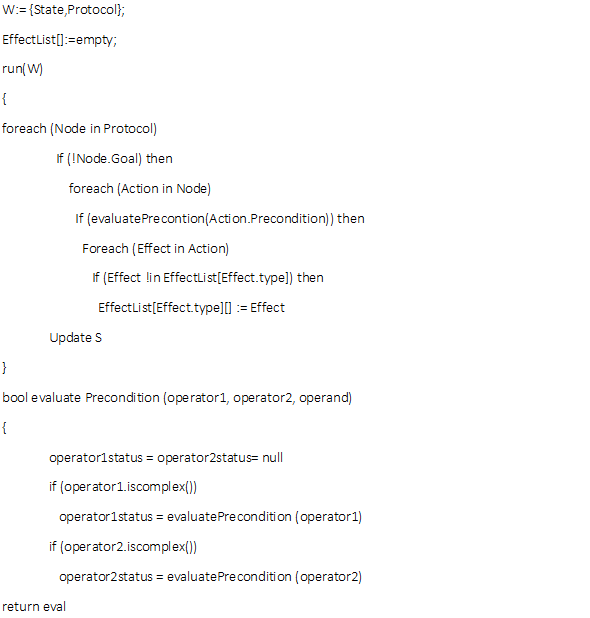
Pseudocode for our knowledge representation.

In the pseudocode of Figure 2 for the XML layer, it can be seen how our interpreter will read the XML file. The structure is designed so that the interpreter will use a small number of recursive functions to read a large amount of code. This can be seen in the pseudocode, where each time an evaluation is found, it calls a function to evaluate the logical value of the conditions.

The rule sets are arranged in a hierarchical structure to capture basic AI planning concepts; inner rules will only evaluate if outer rules in the hierarchy are true. Nodes are aggregates of conditions, which are sets of data as well as a set of rules. Each XML node also has a goal representation, a precondition representation, and an action representation. An XML node corresponds to a basic AI planner module. After the execution of the actions, there will be an update of the representation in terms of state representations, as seen in the pseudocode.

Nodes are the containers where a particular set of goals are established; this is used to give a structure and readability to the XML file.

Goals are considered the main representational purpose for the node to achieve, and when the representation conditions are accomplished, the node will be considered complete and will no longer be able to execute. This is so the system will not compute statements for an undefined period of time. For each of the conditions or rules inside the goal representations and precondition representations, we declare a Boolean expression with the logical operator or operators, including the set operation, in a set as well as in range. Inside each Boolean expression, we reference a represented semantic path to a symbol to access the required values of concept representations that can be related to a specific ID in the database.

Preconditions are the statements that are required to be satisfied before a rule triggers the execution of the associated rule action. Even if the rule inside the goal satisfies the conditions, the action will not be executed. In our knowledge representation, actions can be triggered in 4 different ways: (1) the passage of time; (2) the entry of a data pattern for a patient’s symptom, problem, or diagnosis; (3) the entry of a representation of a diagnostic test value; and (4) the entry of a representation of a treatment result.

Actions are the representation’s output based on specific input conditions, which in turn are based on the rule by which goals and preconditions are satisfied. The actions that are applicable to a state are all those whose preconditions are satisfied. An action means there is a transition between states for the state machine, meaning that certain medical conditions were satisfied in the goal and precondition nodes. An action might be a new request for data, a message, a medical alert, a medical recommendation, or even a calculation, depending on the situation. At this point, it is important to make it clear that when an action takes place, the conditions in the goal will be completed. Otherwise, the node will execute every time the system evaluates the conditions.

The result of our knowledge representation is written and stored in an XML file. XML-encoded rules represent state transitions of the state machine system. This XML file and associated interpreter will be the inference engine core for any expanded future CDSS CIGs with state machine behavior in our future designs.

### XSD Structure

**Figure 3:**
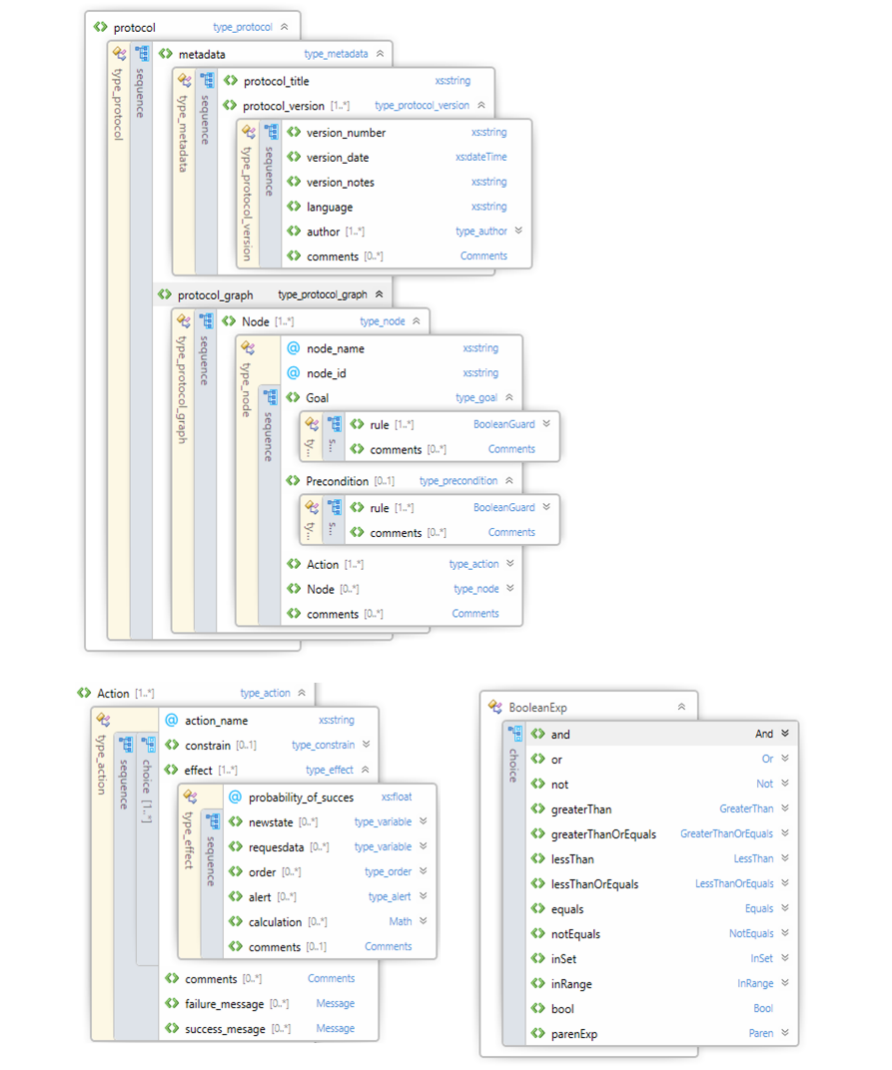
XML Schema Definition file schema.

An XSD schema file was designed with the intention to ease the creation of the XML representations of CPGs with the medical knowledge representation proposed. In Figure 3, the final structure for our knowledge representation is presented. The most important data types of the structure are (1) Boolean expressions, (2) symbols, (3) values, (4) InSets, and (5) Parens.

First, the Boolean expression can be any of the logical operators: “and”, “or”, or “not”. It may also be any of the comparison operators: equal to, greater than, less than, not equal to, greater than or equal to, or less than or equal to. It can be true or false. It also includes the set operations “in-set” and “in-range.”

Second, a symbol is just a string representation of a variable.

Third, a value is the different “values” that symbols can take.

Fourth, the InSet type represents whether a particular symbol is in the set of values. The set can either be empty or infinite.

Fifth, the Paren type represents a parenthesized expression. It contains both the opening and closing parentheses and the expression within the parentheses.

To demonstrate the proposed knowledge representation, we will now show the code of an XML example for the medical situation of choosing a treatment when bacteria are present in the urine. In our example, knowledge is used to suggest a treatment when bacteria are present and their presence is captured in the record. The goal of our example is a patient free of bacteria in the urine. When this is conceptualized as a state machine system, the goal will be achieved by a state machine node representing the patient no longer having the bacteria present in the urine. Our preconditions will be satisfied and rules will be triggered by the colony-forming unit (CFU) value found in a previous urine culture test. There can be multiple kinds of potential actions. We want to address two common actions, a drug order for treatment according to the CFU count and an alert to the physician to determine if there remain bacteria in the urine.

If the patient’s health improves but the lab result indicates there is still the presence of bacteria in the urine, but with a lower CFU, the system should evaluate the conditions and suggest a different treatment more matched to the patient conditions. The goal will still be the same, since the presence of the bacteria is still positive, but the preconditions (trigger) and the actions will be different.

For illustrative purposes, Multimedia Appendix 1 shows the contrast of an Arden medical logic module (MLM) for this task and our XML file representation, both with the same logic of treating a patient with bacterial infection. They share some similarities, as intended in the design structure. In the maintenance section, it is clear that the Arden syntax allows more details, being able to hold information for the guideline’s author, version, institution, validation, and more. Both contain the same encoded statements (medical knowledge), in this case CFUs over 100,000, which trigger the generation of an antibiotic order. A big difference is the existence of the encoded goal in our knowledge representation proposal, where its existence with our state machine behavior makes actions no longer dependent only on the goal itself but also on the current state of the patient. This means that if the same goal is supplied for different patient states, it can lead to different actions, like different treatments or a different message sent to the physician.

## Discussion

### Summary of Findings

In Table 1, we presented a comparison between some common CIGs used for CDSS. Each one has a unique representation language used to encode the medical knowledge. Our approach uses XML in order to take advantage of its flexible format to facilitate the electronic sharing of structured data. More importantly, we can take advantage of its flexibility to standardize CIG structure design in a low-specification language that is ubiquitous, universal, and human readable.

Our plans involve the computerization of antenatal health care guidelines with our knowledge representation for a web-based CDSS, and then a mobile app. The clinical decisions suggested will assist clinicians in three situations. First, it will evaluate obstetrical risk and inform clinicians and raise health alerts about patients’ medical conditions, like pre-eclampsia, diabetes, or premature birth. Second, it will suggest medical procedures and drugs according to the gestational age of the mother and her clinical condition. Third, it will make referral suggestions for transferring patients from primary to secondary care and vice versa.

As discussed in the Introduction, our knowledge representation was designed for an antenatal care CDSS project with requirements for both a web and mobile CDSS but also designed to be usable for many other purposes as part of future implementations of other clinical applications. Future applications will test the adequacy of the framework to support additional information and workflow needs of CPGs for other health services and allow us to expand the framework as needed.

For our particular antenatal domain problem scenarios, we found our current framework and implementation methods adequate to capture the workflow and decision logic of the existing guidelines (Table 2). The domain knowledge, task knowledge, and inferences required were easily extracted and separable into the architecture components and the underlying representation. We did not find it necessary to use a more complex representation, such as the alternatives discussed earlier in the paper (Table 1). Adequate domain object representations were accommodated in the framework as well as the representation of the task ontology we used. The inferences required fit into the simple rule syntax and the deterministic state change representations in the state machine conceptualization. The inferences could also easily be separated in the modular structure of the described state machine, both hierarchically and by arranging rule application precedence. For the domain ontology and task ontology in particular, we did not need a more complex hierarchical ontology, such as those in published biomedical ontologies. Semantic elements such as object properties and instances were captured in a straightforward manner in our XML framework.

It is possible to accommodate either controlled vocabulary terms from standardized sources in the XML representation or ad hoc terms. We anticipate that controlled vocabulary terms will be needed for more complex systems for interoperability with other care systems or to accommodate a greater variety of users than our current system allows. Standardized terms would also result in a more understandable human-computer interface. In more complex systems, there is often a need to include common terms for the purpose of human and machine reference and communication, but with minimal addition, the current approach appears to accommodate any Arden syntax medical logic module representation.

The task modeling ontology we chose was also adequate for our current system and easily represented in XML. The elements of the state machine process representation for the system’s problem-solving steps that achieve a task goal and decompose tasks into subtasks were adequate for the antenatal system. A stochastic process representation addition is anticipated for other domains but can be accompanied within a stochastic state machine framework. The rule representation used also proved adequate for capturing the performance of Arden syntax MLMs.

An important step to consider in future projects will be parsing existing Arden syntax MLMs to our representations, since Arden syntax is an official and commonly used standard and there are plenty of MLMs with encoded medical knowledge using it. Certainly, at its current stage of development, our approach is not able to capture the more sophisticated logic required by the axioms using semantic web rules, such as Semantic Web Rule Language [34], which is commonly used in Web Ontology Language–based CDSSs.

We have sacrificed generality in the representation to achieve a minimal design footprint and execution efficiency across multiple computing environments while adequately capturing the guidelines required for our project. It is possible to think of the current representation as a compiled or low-level interpreted version of a more complex representation of guidelines that retains the computation resource efficiency and probability of the current approach for both web applications and mobile apps. In that case, an associated higher-level representation allowing for more generality and a friendlier user development environment based on the more complex representations would be possible.

In the future, having an interface design tool for authoring and editing our XML-represented protocols will be useful to ease the encoding of knowledge. XML is not hard to understand once people get used to it, but this does not mean that there cannot be another tool to encode medical knowledge more easily with XML. A graphic user interface that is object oriented can likely be designed to help the encoding process for health care professionals, who are more motivated than computer engineers to participate in encoding medical knowledge.

### Conclusions

Usability of a CDSS depends heavily on the match of system flow to health care workflow. Allowing for consistent stepwise processing of health data over time can support adherence to best clinical practice. Consistent with best-practice CPGs, the intent of the CDSS is to reduce the caregiver’s mental load and prevent possible errors in clinical tasks that involve the analysis of a patient’s status and the use of this context for action decisions. Our knowledge representation framework incorporates fundamental elements of other CIGs used in CDSSs in medicine to encode a number of antenatal health care CPGs and associated clinical workflows. The framework appears general enough to be useful with other CPG-to-CIG projects in medicine.

XML proved to be a language expressive enough to describe the planning problems in a computable form and both restrictive and expressive enough to implement in a clinical system. It can be effective for mobile apps, where intermittent communication requires a small-footprint autonomous app. It can be used to incorporate overlapping capabilities of more specialized CIGs in medicine. These qualities of the XML language give it viability for use in CDSSs as a knowledge engine core that is based on a widely available and understood collection of technologies for web applications and mobile apps.

## Appendix

Multimedia Appendix 1 Example: comparison between Arden syntax and our XML file in starting antibiotic treatment.

## Abbreviations

AI: artificial intelligence
CDSS: clinical decision support system
CFU: colony-forming unit
CIG: computer-interpretable guidelines
CPG: clinical practice guideline
FCM: fuzzy cognitive map
MLM: medical logic module
MVC: model-view-controller
XSD: XML Schema Definition
