# Supplementary material for: XML Data and Knowledge-Encoding Structure for a Web-Based and Mobile Antenatal Clinical Decision Support System: Development Study
Source: JMIR Form Res. 2020 Oct 16;4(10):e17512. doi: 10.2196/17512 (PMC7600017; doi:10.2196/17512)
Supplement: Multimedia Appendix 1 [file formative_v4i10e17512_app1.docx]

**Multimedia Appendix 1.** Example: comparison between Arden syntax and our XML file in starting antibiotic treatment.

| **Arden syntax** | **XML** |
| --- | --- |
| **maintenance:**   title: Screen for presence of bacterial infection   and pharmacy order for an antibiotic;;  filename: bacterial-treatment;;  version: 1.0;;  institution: Universidad Pontificia Bolivariana;;  author: Sebastian Uribe, Eng;;  specialist: ;;  date: 2017-03-07T00:00:00 ;;  validation: production;;  **library:**   **purpose:** To determine if a patient has both laboratory evidence of bacterial infection and an active order for an antibiotic;;   **explanation:** the antibiotic must be adjusted when it is administered to a patient who has an infection. This module sends an alert if one of this class of antibiotics is ordered for a patient who has laboratory evidence of bacterial infection to help ensure that appropriate action (e.g., dosage adjustment) is taken if needed. ;;   **keywords:** bacterial infection, antibiotic;;  citations: ;; | <metadata xmlns="">  <protocol_title>BacteriaTreatment</protocol_title>  <protocol_version>  <version_number>1</version_number>  <version_date>20170307T00:00:00</version_date>  <version_notes>Screen for presence of bacterial infection and pharmacy order for an antibiotic </version_notes>  <language>en</language>  <author>  <author_name>Sebastian Uribe</author_name>  <author_filiation>Universidad Pontificia Bolivariana</author_filiation>  </author>  </protocol_version>  </metadata> |
| **Not in Arden syntax** | <Goal>  <rule>  <booleanExp>  <equals>  <symbol>  <SymbolPath> ClinicalUrineTest.LabResult.last </SymbolPath> <CommentSymbol>Patient</CommentSymbol>  </symbol>  <value>Positive</value>  </equals>  </booleanExp>  </rule>  </Goal> |
| **knowledge:**   **type:** data-driven;;   **data:**  /* evoke on storage of a pharmacy order */  storage_of_antibiotic_order := event  {'30343','30345';'30343','30346'};  /* read the antibiotic order that evoked the MLM */  antibiotic_order := read last  {'evoking','dam'="PDQORD1",'auxstr'="0013",  'constraints'="C****",'status_value'="A",  'display_header'="R",'display_comp'="V"; ; '23946'};  urine_test := last(CFU where they are number);  CFU_boundary := 100000; /* upper reference range value */ ;; | <Precondition>  <rule>  <booleanExp>  <or>  <booleanExp1>  <greaterThan>  <symbol>  <SymbolPath>ClinicalUrineTest.CFU-Result.last</SymbolPath>  <CommentSymbol></CommentSymbol>  </symbol>  <value>100000</value>  </greaterThan>  </booleanExp1>  <booleanExp2>  <notEquals>  <symbol>  <SymbolPath>Drug.order. Antibiotic name</SymbolPath>  <CommentSymbol>Check existence of order that can provide data </CommentSymbol>  </symbol>  <value>NULL</value>  </notEquals>  </booleanExp2>  </or>  </booleanExp>  </rule>  </Precondition> |
| **evoke:**  /* evoke on storage of a pharmacy order */  storage_of_antibiotic_order;;   **logic:**  if (antibiotic_order is null) or  (CFU is null) then /* insufficient data */  conclude false;  end if;  /* check marker for bacterial infection */  if CFU <= CFU_boundary then  conclude false;  end if;  /* otherwise CFU is high and pt is on Infection */  conclude true; ;;  **action:**   write "The patient has laboratory evidence of bacterial " \|\| "infection (CFU " \|\|" on " \|\| time of urine test \|\| "). An active " \|\| "order for an antibiotic has been " \|\| "recorded. Such antibiotics may cause or worsen bacterial " \|\| "infection, and special dosing may be required. " \|\|  "This may not be applicable to topical preparations. " \|\|  "Appropriate action should be taken as needed."; ;; end: | <Action>  <effect>  <order>  <order_name>  <SymbolPath>Observation.DrugTreatment</SymbolPath>  <CommentSymbol>The patient has laboratory evidence of bacterial infection  </CommentSymbol>  </order_name>  <drugs>  <SymbolPath>Drug.AntibioticName</SymbolPath>  <CommentSymbol>An active order for an antibiotic has been recorded. </CommentSymbol>  </drugs>  <Comments></Comments>  </order> </effect>  <effect>  <alert>  <channel>  <browser>  <recipient>Attending_professional</recipient>  <subject>Bacteria Presence Positive</subject>  </browser>  </channel>  <content>The patient has laboratory evidence of bacterial infection Also, an active order for an antibiotic has been recorded. Such antibiotics may cause or worsen bacterial. infection, and special dosing may be required. This may not be applicable to topical preparations. Appropriate action should be taken as needed. </content>  <domain>  <Actor></Actor>  </domain>  </alert>  </effect>  </Action> |
